# Rationale and design of an investigator-initiated, multicenter, prospective, placebo-controlled, double-blind, randomized trial to evaluate the effects of finerenone on vascular stiffness and cardiorenal biomarkers in type 2 diabetes and chronic kidney disease (FIVE-STAR)

**DOI:** 10.1186/s12933-023-01928-y

**Published:** 2023-07-31

**Authors:** Atsushi Tanaka, Hirotaka Shibata, Takumi Imai, Hisako Yoshida, Motoaki Miyazono, Naohiko Takahashi, Daiju Fukuda, Yosuke Okada, Hiroki Teragawa, Satoru Suwa, Keisuke Kida, Masao Moroi, Isao Taguchi, Shigeru Toyoda, Michio Shimabukuro, Kengo Tanabe, Kenichi Tanaka, Masaomi Nangaku, Koichi Node, the FIVE-STAR trial investigators

**Affiliations:** 1 Department of Cardiovascular Medicine, Saga University, Saga, Japan; 2 Department of Endocrinology, Metabolism, Rheumatology and Nephrology, Faculty of Medicine, Oita University, Yufu, Japan; 3 Department of Medical Statistics, Osaka Metropolitan University Graduate School of Medicine, Osaka, Japan; 4 Department of Nephrology, Saga University, Saga, Japan; 5 Department of Cardiology and Clinical Examination, Oita University, Yufu, Japan; 6 Department of Cardiovascular Medicine, Osaka Metropolitan University Graduate School of Medicine, Osaka, Japan; 7 First Department of Internal Medicine, University of Occupational and Environmental Health, Kitakyushu, Japan; 8 Department of Cardiovascular Medicine, JR Hiroshima Hospital, Hiroshima, Japan; 9 Department of Cardiology, Juntendo University Shizuoka Hospital, Izunokuni-Shi, Japan; 10 Department of Pharmacology, St. Marianna University School of Medicine, Kawasaki, Japan; 11 Department of Cardiovascular Medicine, Toho University Ohashi Medical Center, Tokyo, Japan; 12 Department of Cardiology, Dokkyo Medical University Saitama Medical Center, Saitama, Japan; 13 Department of Cardiovascular Medicine, Dokkyo Medical University School of Medicine, Mibu, Japan; 14 Department of Diabetes, Endocrinology, and Metabolism, Fukushima Medical University, Fukushima, Japan; 15 Division of Cardiology, Mitsui Memorial Hospital, Tokyo, Japan; 16 Wakamatsu Hospital of the University of Occupational and Environmental Health, Japan, Kitakyushu, Japan; 17 Division of Nephrology and Endocrinology, The University of Tokyo Graduate School of Medicine, Tokyo, Japan

**Keywords:** Finerenone, Vascular stiffness, Biomarker, Type 2 diabetes, Chronic kidney disease

## Abstract

**Background:**

The overactivation of mineralocorticoid receptor (MR) plays a key pathological role in the progression of cardiovascular and renal diseases by promoting pro-inflammatory and pro-fibrotic signaling. Recently, it has been found that finerenone, a novel nonsteroidal selective MR antagonist, can robustly improve cardiorenal outcomes in patients with type 2 diabetes (T2D) and a wide spectrum of chronic kidney disease (CKD). However, the mechanisms underlying the cardiorenal benefits of finerenone are poorly understood. Further, whether the clinical benefits are mediated by an improvement in vascular stiffness is not confirmed. Therefore, the current study aims to evaluate the effects of finerenone on vascular stiffness as assessed using cardio ankle vascular index (CAVI) and relevant cardiorenal biomarkers in patients with T2D and CKD.

**Methods:**

The Effects of Finerenone on Vascular Stiffness and Cardiorenal Biomarkers in Type 2 Diabetes and Chronic Kidney Disease (FIVE-STAR) is an ongoing, investigator-initiated, multicenter, prospective, placebo-controlled, double-blind, randomized clinical trial in Japan. Its target sample size is 100 subjects. Recruitment will be performed from September 2023 to July 2024. After obtaining informed consent, eligible participants with T2D and CKD (25 mL/min/1.73 m^2^ ≤ estimated glomerular filtration ratio [eGFR] < 90 mL/min/1.73 m^2^ and 30 mg/g Cr ≤ urinary albumin-to-creatinine ratio [UACR] < 3500 mg/g Cr) will be equally randomized to receive 24-week treatment with either finerenone (starting dose at 10 mg once daily in participants with a baseline eGFR < 60 mL/min/1.73 m^2^ or at 20 mg once daily in those with a baseline eGFR ≥ 60 mL/min/1.73 m^2^) or dose-matched placebo. The primary endpoint is the change from baseline in CAVI at 24 weeks. The secondary endpoints are changes from baseline in UACR at 12 and 24 weeks and relevant serum and urinary biomarkers at 24 weeks. As an exploratory endpoint, proteomic analysis using the Olink® Target 96 panels will be also performed.

**Discussion:**

FIVE-STAR is the first trial evaluating the therapeutic impact of finerenone on vascular stiffness and relevant cardiorenal biomarkers in patients with T2D and CKD. This study will provide mechanistic insights on the clinical benefits of finerenone based on recent cardiovascular and renal outcome trials.

*Trial registration* Unique Trial Number, NCT05887817 (https://clinicaltrials.gov/ct2/show/NCT05887817) and jRCTs021230011 (https://jrct.niph.go.jp/latest-detail/jRCTs021230011).

**Supplementary Information:**

The online version contains supplementary material available at 10.1186/s12933-023-01928-y.

## Background

Type 2 diabetes (T2D) is a major cause of chronic kidney disease (CKD) and end-stage renal disease, which pose significant economic and medical burden on worldwide health care [1]. T2D and CKD are closely and synergistically associated with a high risk of cardiovascular events, including heart failure (HF), and mortality [2]. A recent international guideline for patients with T2D and CKD recommends a comprehensive approach for achieving healthy lifestyle and preventing risk factors with pharmacotherapy, which can improve kidney and cardiovascular outcomes [3]. Regarding the renoprotective effects of pharmacotherapy, in addition to blood pressure optimization with the conventional use of renin-angiotensin system (RAS) blockers, such as an angiotensin-converting enzyme inhibitor and angiotensin-receptor blocker, sodium-glucose cotransporter 2 (SGLT2) inhibitors have been recently found to improve cardiovascular and renal outcomes in patients with CKD, irrespective of diabetes status [4–6]. Moreover, it is recommended for reducing the risk of the aforementioned events in patients with CKD [3, 7]. However, the risk of CKD progression and cardiovascular events remains high, thereby indicating the presence of untreated and residual risk, which should be assessed.

Accumulated evidence suggests that a high plasma aldosterone concentration and mineralocorticoid receptor (MR) overactivation play a pathophysiological role in metabolic syndromes and several cardiorenal diseases via pro-inflammatory and pro-fibrotic signaling-mediated injuries in the tissues and organs [8, 9]. Previous meta-analysis showed that steroidal MR antagonists (MRAs) reduced urinary protein levels and delayed CKD progression [10]. In addition, the use of these drugs is currently among the evidence-based guideline-directed medical therapies in symptomatic patients with HF with reduced ejection fraction (HFrEF) [11].

Finerenone is a novel nonsteroidal MRA that has a greater MR selectivity and affinity than steroidal MRAs [12]. In the recent landmark trials (Finerenone in Reducing Kidney Failure and Disease Progression in Diabetic Kidney Disease [FIDELIO-DKD] and Finerenone in Reducing Cardiovascular Mortality and Morbidity in Diabetic Kidney Disease [FIGARO-DKD]) [13, 14], treatment with finerenone reduced the risks of CKD progression and cardiovascular events in patients with T2D across a broad spectrum of patients with CKD who had been receiving standard therapy with RAS blockers [15]. These findings emphasize the promising therapeutic role of finerenone in improving cardiovascular and kidney outcomes in patients with T2D and CKD. However, the mechanisms underlying the abovementioned clinical benefits of finerenone are poorly understood.

Previous data have supported the notion that high vascular stiffness is closely associated with the development of CKD and the risk of cardiovascular events in various populations, including patients with T2D [16]. To test our hypothesis that the cardiorenal benefits of finerenone can be mediated by improvement in vascular stiffness and to further explore the cardiovascular and renal actions of finerenone, this mechanistic clinical trial aims to evaluate the effects of finerenone versus placebo on vascular stiffness as assessed using cardio ankle vascular index (CAVI) and relevant cardiorenal surrogate biomarkers in patients with T2D and CKD.

## Methods

### Trial overview, ethical consideration, and patient recruitment

The Effects of Finerenone on Vascular Stiffness and Cardiorenal Biomarkers in Type 2 Diabetes and Chronic Kidney Disease (FIVE-STAR) is an ongoing, investigator-initiated, multicenter, prospective, two-arm parallel, placebo-controlled, double-blind, randomized clinical trial currently performed in 13 sites in Japan (Additional file 1). The SPIRIT checklist is provided in Additional file 2. This investigator-initiated research program was approved and granted by Bayer Yakuhin, Ltd. (Osaka, Japan). However, this funding agency has no role in the study execution and reporting.

The study protocol was approved by the Certified Review Board of Fukushima Medical University (no. F2023001), and the protocol amendments will be reviewed in each case. Currently, it is conducted in accordance with the Declaration of Helsinki and the Clinical Trial Act in Japan. The trial is registered in NCT05887817 (https://clinicaltrials.gov/ct2/show/NCT05887817) and jRCTs021230011 (https://jrct.niph.go.jp/latest-detail/jRCTs021230011). Trial recruitment will begin in September 2023 and is expected to end by July 2024. After the patients will be initially screened for eligibility based on their previous medical records, they will receive adequate explanations about the trial plan by investigators before they provide a written informed consent.

### Study population

The target study population includes patients with T2D and CKD. Table 1 shows the detailed inclusion and exclusion criteria. In brief, patients aged ≥ 20 years who were diagnosed with T2D (HbA1c < 10.0%) and CKD (25 mL/min/1.73 m^2^ ≤ estimated glomerular filtration ratio [eGFR] < 90 mL/min/1.73 m^2^ and 30 mg/g Cr ≤ urinary albumin-to-creatinine ratio [UACR] < 3500 mg/g Cr) and who did not present with changes in medication status for T2D and CKD within four weeks prior to providing consent were eligible for the analysis. Patients with several prespecified medical conditions, such as hyperkalemia (serum potassium ≥ 4.9 mEq/L), symptomatic HFrEF (left ventricular ejection fraction [LVEF] ≤ 35%), uncontrolled hypertension, and those with a recent history of cardiovascular and renal events, will be excluded from the trial.

**Table 1:** Inclusion and exclusion criteria

Inclusion criteria	Exclusion criteria
1. Patients who provided a written informed consent 2. Patients who are aged ≥ 20 years at the time of consent (regardless of sex) 3. Patients with T2D 4. Patients with chronic kidney disease who meet the following criteria: i) eGFR ≥ 25 mL/min/1.73 m^2^ and < 90 mL/min/1.73 m^2^ ii) UACR ≥ 30 mg/g Cr and < 3500 mg/g Cr 5. Patients who did not change their medications for T2D and CKD within the last 4 weeks prior to obtaining consent	1. Patients who are currently taking or have taken MRAs containing finerenone within the last 4 weeks prior to obtaining consent 2. Patients with a history of finerenone hypersensitivity 3. Patients with an HbA1c level of > 10% 4. Patients with a serum potassium level of ≥ 4.9 mEq/L 5. Patients with NYHA class II–IV HFrEF (LVEF ≤ 35%) 6. Patients with poorly controlled hypertension (e.g., systolic BP ≥ 170 mmHg, diastolic BP ≥ 110 mmHg, or hypertensive emergencies) 7. Patients with a history of ischemic stroke, acute coronary syndrome, cardiovascular surgery or percutaneous intervention, or hospitalization for worsening heart or renal failure within the last 8 weeks prior to obtaining consent 8. Patients with a preplanned surgical or percutaneous intervention for coronary artery reconstruction or other cardiovascular diseases during the individual observation period 9. Patients with a preplanned treatment such as electrical cardioversion, cardiac resynchronization therapy, and pacemaker implantation during the individual observation period 10. Patients with preplanned dialysis or kidney transplantation during the individual observation period 11. Patients with severe hepatic dysfunction (Child–Pugh class C) 12. Patients receiving itraconazole, ritonavir-containing products, atazanavir, darunavir, fosamprenavir, cobicistat-containing products, or clarithromycin 13. Patients with Addison’s disease 14. Patients with active infectious diseases 15. Pregnant, possibly pregnant, or lactating patients 16. Other patients deemed inappropriate for this study by the investigators (e.g., those with renal artery stenosis, one kidney, or active malignancy)

BP, blood pressure; CKD, chronic kidney disease; eGFR, estimated glomerular filtration rate; HFrEF, heart failure with reduced ejection fraction; LVEF, left ventricular ejection fraction; MRA, mineralocorticoid receptor antagonist; NYHA, New York Heart Association; T2D, type 2 diabetes; UACR, urinary albumin-to-creatinine ratio

### Randomization and blinding

All eligible subjects will be randomly assigned (1:1) either to the finerenone or placebo group via a web-based program with dynamic allocation using a minimization method balanced by age (< 70, ≥ 70 years), sex (female, male), eGFR (< 45, ≥ 45 mL/min/1.73 m^2^), and use of SGLT2 inhibitor (yes, no) at the time of screening.

This trial will be conducted in a double-blind manner. After randomization, the participants, attending physicians, and other individuals involved in the trial will be masked to the group allocation results until data fixation is complete. The person (HY) responsible for allocation shall open (non-blinding) the result after the database is fixed. Blinding codes will be opened only in urgent situations (e.g., when patients require appropriate medical attention due to illness, and when safety of the participants should be ensured). If opened, the principal investigator will be notified immediately, and the participant will be withdrawn from the study.

### Treatment and follow-up

**Fig. 1:**
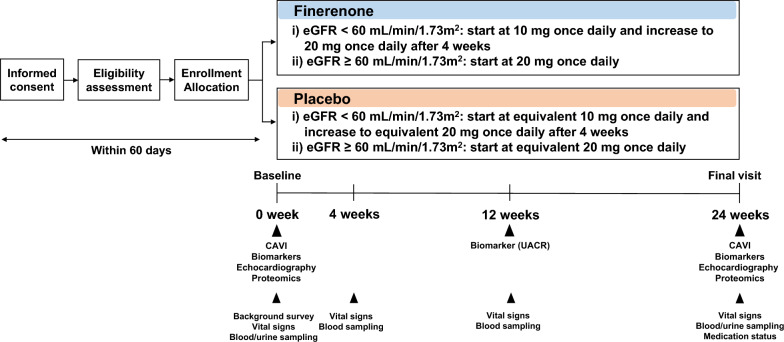
Study design. CAVI, cardio ankle vascular index; eGFR, estimated glomerular filtration rate; T2D, type 2 diabetes; UACR, urinary albumin-to-creatinine ratio. 1 If the date of discontinuation is within the allowance of each visit, observation and examination will be performed to the extent possible. 2 In principle, baseline testing should be conducted within 60 days of obtaining consent, and study drug dosing should begin thereafter. 3 Height, weight, office and home blood pressure (optional), and office and home pulse rate (optional). Data after obtaining consent will be prioritized. However, data within 3 months prior to obtaining consent can be used. Height will be measured at baseline only. 4 Serum creatinine and eGFR alone. 5 Serum pentosidine, urinary type IV collagen, urinary α1-microglobulin, β2-microglobulin, urinary neutrophil gelatinase-associated lipocalin, N-acetyl-β-d-glucosaminidase, and urinary liver-type fatty acid-binding protein corrected by urinary creatinine. 6 Hemoglobin, hematocrit, serum albumin and potassium, HbA1c, plasma (or serum) aldosterone concentration, and plasma (or serum) renin activity (or concentration). 7 Serum potassium alone. 8 Serum potassium and HbA1c alone. 9 Serum potassium, HbA1c, plasma (or serum) aldosterone concentration, and plasma (or serum) renin activity (or concentration) alone

All participants will visit their regular physicians for usual health care, individualized background medications, administration of study drug, and monitoring of safety, and drug adherence during the study. In principle, baseline tests should be conducted within 60 days after obtaining consent, and treatment with study drug should begin thereafter. To assess the study endpoints, post-randomization follow-up visits are scheduled at 4 and 12 weeks before the final visit at 24 weeks (Fig. 1).

All randomized participants will be instructed to take either dose-adjusted finerenone or placebo orally once daily (preferably at the same time each day in the morning). Participants with a baseline eGFR < 60 mL/min/1.73 m^2^ will receive finerenone at a starting dose of 10 mg once daily or dose-matched placebo. In accordance with the information in the latest package insert of finerenone, the dose will be increased to 20 mg once daily in both groups at 4 weeks after the initiation of the first dose. Meanwhile, participants with a baseline eGFR ≥ 60 mL/min/1.73 m^2^ will receive finerenone at starting dose of 20 mg once daily or dose-matched placebo. After starting or increasing the finerenone dose, the dose can be reduced, or the drug can be discontinued according to the individual medical situations (including changes in serum potassium levels and eGFR) (Additional file 3), based on the judgment of local investigators. In both groups, there will be no restrictions on the use of other drugs for T2D, CKD, and other comorbidities. However, no new administration of a SGLT2 inhibitor will be implemented during the observation period. In addition, the dosage, and administration of background medications will not be changed. However, changes based on the discretion of local investigators will be permitted according to the medical condition of the participants. Table 2 shows the criteria on study discontinuance. If the date of discontinuance is within the allowance of each visit, the relevant examinations will be performed to the extent possible.

**Table 2:** Discontinuance criteria

6. If a participant declines to participate in the trial or withdraws his/her consent
7. If a participant finds it challenging to visit the research institution due to relocation or hospital transfer
8. If participants did not meet the selection criteria or if they have violated the inclusion/exclusion criteria after the initiation of the study
9. If a participant is unable to continue the research due to the development of an adverse event according to the investigator’s discretion
10. If the investigator finds it challenging to continue the research due to worsening of the primary disease or complications
11. If the blind code is opened 12. If the investigator finds that it is appropriate to discontinue participation in the trial due to certain reasons

### Measurements and endpoints

**Fig. 2:**
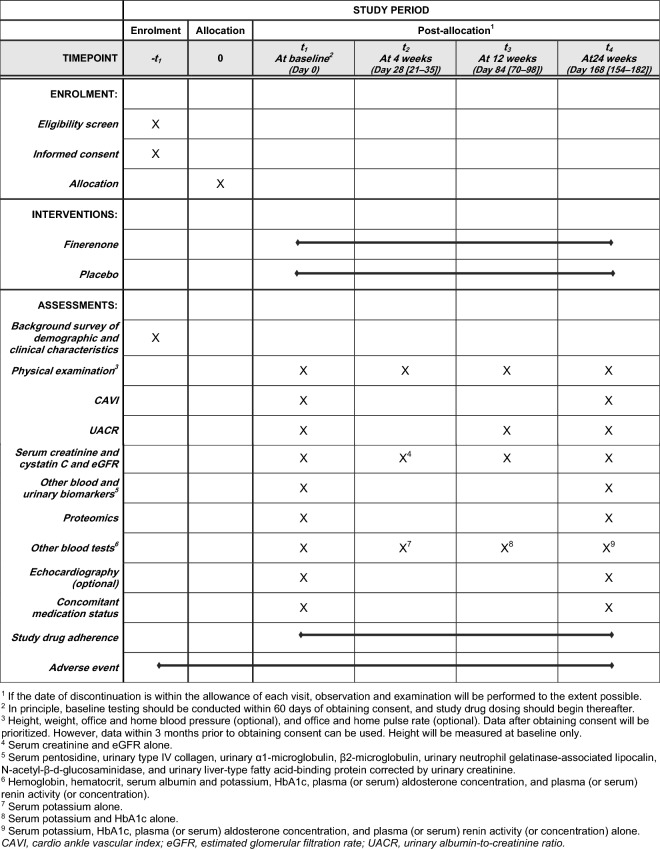
SPIRIT flow diagram

The SPIRIT flow diagram is shown in Fig. 2. The primary endpoint in this trial is the change from baseline in CAVI at 24 weeks after the initiation of protocol treatment. The secondary endpoints include (1) the proportional changes from baseline in the geometric means of UACR at 12 and 24 weeks post-protocol treatment (key secondary endpoint) and (2) the proportional changes from baseline in the geometric means of serum pentosidine, urinary type IV collagen, urinary α1-microglobulin, β2-microglobulin, urinary neutrophil gelatinase-associated lipocalin, N-acetyl-β-d-glucosaminidase, and urinary liver-type fatty acid-binding protein levels corrected by urinary creatinine at 24 weeks post-protocol treatment. The other efficacy endpoints include changes from baseline in (a) vital signs including body weight, body mass index, estimated extracellular volume calculated using the body surface area, office and home blood pressure, pulse pressure, and heart rate at 4, 12, and 24 weeks post-protocol treatment; (b) laboratory measures including eGFR, serum creatinine, cystatin C*, and potassium, HbA1c*, and plasma (or serum) aldosterone concentration**, and plasma (or serum) renin activity (or concentration)** at 4, 12, and 24 weeks post-protocol treatment (*12 and 24 weeks only; **24 weeks only); (c) augmentation index (AI) and percent mean arterial pressure (%MAP) simultaneously measured by CAVI at 24 weeks post-protocol treatment; and (d) cardiac function indices (left ventricular ejection fraction, septal e′, lateral e′, E, E/e′, left ventricular mass index, left atrial dimension, and left atrial volume index) assessed on echocardiography at 24 weeks post-protocol treatment. In addition, as an exploratory endpoint, proteomic analysis using the Olink^**®**^ Target 96 panels using high-multiplex immunoassays will be performed at baseline and 24 weeks.

Data collection and management will be performed via an electronic data capture system (eClinical Base, Translational Research Center for Medical Innovation, Kobe, Japan). Personal information about participants will be masked and coded. Data monitoring will be conducted independently (The Organization for Clinical Medicine Promotion, Tokyo, Japan).

### CAVI measurement

Vascular (arterial) stiffness will be assessed as CAVI automatically measured using the VaSera device (Fukuda Denshi, Co., Ltd., Tokyo, Japan) at baseline and 24 weeks. Detailed rationale and CAVI examination method have been described elsewhere [17]. After a few minutes of rest in the supine position, CAVI measurement will be conducted using a standard protocol according to the manufacture’s instruction. ECG electrodes will be placed on both wrists, a microphone on the sternum for detecting heart sounds, and cuffs on the upper arms and ankles in the supine position. CAVI will be automatically determined using the following formula [18]: CAVI = a {(2ρ/∆P) × ln (Ps/Pd) PWV^2^} + b, where a and b are constants for converting the CAVI values, ρ is blood density, ΔP is Ps (systolic blood pressure)–Pd (diastolic blood pressure), and PWV is the pulse wave velocity. The average of the left and right sides of CAVI will be used in the analysis. AI and %MAP will be simultaneously and automatically measured using the device.

### Safety

Throughout the study, safety information will be collected from all participants by recording adverse events (AEs), including hyperkalemia, irrespective of severity, and their causal associations to the trial drugs and protocol. In cases of AEs, local investigators must assess the severity or grade of AEs, procedures conducted, outcomes, and their associations to finerenone. In addition, local investigators must immediately report AEs to the secretariat who, in turn, will promptly report to the Certified Review Board of Fukushima Medical University in accordance with the Clinical Trial Act in Japan, if needed.

### Statistical considerations

#### Sample size estimation

During study planning and preparation, there was no sufficient clinical evidence on the effects of finerenone on vascular stiffness and other cardiovascular and renal surrogate markers in patients with T2D and CKD. Recently, a study with other MRA (Mineralocorticoid Receptor Antagonists in Type 2 Diabetes: MIRAD) showed that treatment with eplerenone for 26 weeks, compared with placebo, significantly improved cardiac structural parameters as assessed by cardiac magnetic resonance and reduced some relevant biomarker levels in patients with T2D [19]. In that study, the total number of participants was 104, thereby indicating the possibility that the interventional effects of MRA on cardiovascular surrogate markers could be detectable in such a sample size scale. As for arterial stiffness as assessed by CAVI, previous studies on patients with T2D and the general population showed that CAVI values increased by 0.5 after approximately 10 years of aging [20, 21]. Thus, lowering CAVI by about 1.0 can be equivalent to delaying the progression of vascular stiffness by approximately 20 years, which would be clinically meaningful. Based on the baseline data of our previous PROTECT study on patients with T2D, but not limited to those with CKD [22], the mean CAVI at baseline was about 9.0, with a standard deviation (SD) of approximately 1.5 (unpublished data). When using that value as a reference, lowering CAVI by 1.0 can correspond to CAVI with an SD of approximately 0.6. Furthermore, a recent secondary analysis of the EMPA-TROPISM study on non-diabetic patients with HFrEF showed that treatment with empagliflozin, a sodium-glucose cotransporter 2 inhibitor, for six months significantly improved aortic stiffness as assessed by PWV relative to placebo (− 0.58 cm/s [SD: − 0.36] vs + 0.60 cm/s [SD: + 0.60 SD]. The estimated effect size is approximately − 0.96 SD, and such a treatment reduced the levels of some inflammatory biomarkers as assessed via proteomics analysis [23]. Although there are no data directly comparing the clinical efficacy of finerenone and empagliflozin at the time of study designing, we hypothesized that finerenone can improve vascular stiffness similar to empagliflozin according to the EMPA-TROPISM trial. Then, we conservatively evaluated that CAVI in the finerenone group can be reduced by at least − 0.6 SD (63% of the effect size detected in the EMPA-TROPISM study) compared with the placebo, with a two-sided significance level of 5%, power of 80%, and drop-out rate of 10%. Finally, the minimum number of participants required to detect a significant difference between the two groups was set at 100 (n = 50 per arm).

#### Statistical analysis plan

Descriptive statistics will be used to summarize all baseline characteristics with the number of participants with data (all variables), mean, standard deviation, median, interquartile range, minimum and maximum (continuous variables), and frequencies and proportions (categorical variables).

Analyses of the efficacy endpoints will be performed on an intention-to-treat-basis, and full analysis set (FAS) will be identified from all randomized participants by excluding participants who (1) withdrew their consent after enrollment, (2) those who are found to be not eligible after enrollment, (3) those have not received any protocol treatment after allocation, and (4) those without any efficacy data after the initiation of protocol treatment. In the FAS population, the participants without serious protocol deviations will be designated as per protocol set for the supplemental efficacy analysis. Regarding the primary endpoint, changes from baseline in CAVI to 24 weeks after protocol treatment will be analyzed using a linear regression model with the treatment group as a fixed effect and the baseline value as a covariate. Mean changes in both treatment groups with two-sided 95% confidence intervals (CIs) will be estimated based on the least squares mean at 24 weeks. Moreover, the group-difference with 95% CI and *P* value will be estimated as a treatment effect. Regarding the secondary endpoints, changes in log-transformed values from baseline to each visit will be estimated using the mixed-effects models for repeated measures with the treatment group, time point, and their interaction as fixed effects and corresponding baseline value as a covariate. The analysis outputs will be obtained by transforming to the original value scale. Further, the proportional changes from baseline in geometric mean with 95% CI in both treatment groups at each visit and their group-ratio with 95% CI as treatment effects will be estimated. Other efficacy endpoints will also be analyzed similarly, as appropriate.

Safety analysis will be conducted on all randomized patients who have received at least one dose of the protocol treatment. The reported AEs should be assigned with a lower-level term code using the Japanese version of the MedDRA/J dictionary of regulatory terms. AEs occurring after the start of protocol treatment will be counted, and AEs that are considered to be causally “related” to finerenone will be considered as its side effects. If an adverse event of the same preferred term occurs more than once in a participant, the number of occurrences, and the number of participants will be counted separately. For all AEs and side effects, the number of occurrences, number of participants, and its percentage with 95% CI in each treatment group will be calculated.

The principal investigator (KN) and a statistician (TI) will finalize the detailed statistical analysis plan before the database lock. All *P* values are two-sided, and a *P* value of < 0.05 indicate statistically significant difference. This trial did not plan interim analysis. Except for proteomics analysis, adjustments for multiple comparisons are not made. A statistician (TI) will have access to the final dataset of the trial. All statistical analyses will be performed using SAS version 9.4 or higher (SAS Institute, Cary, NC, the USA).

## Discussion

**Fig. 3:**
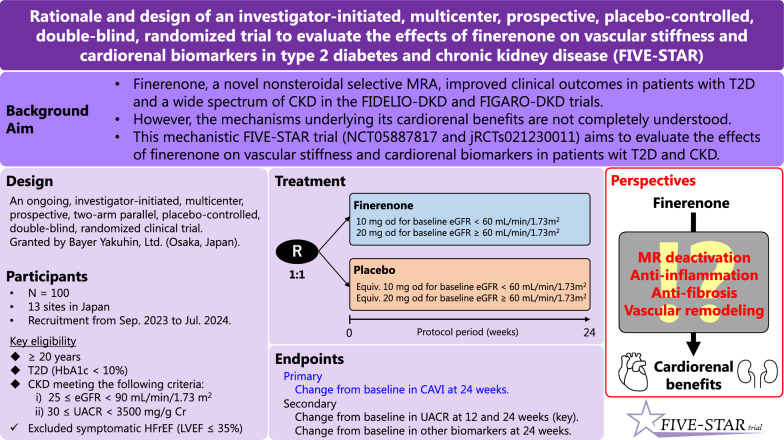
Graphical abstract of the FIVE-STAR trial. CAVI, cardio ankle vascular index; CKD, chronic kidney disease; eGFR, estimated glomerular filtration rate; HFrEF: heart failure with reduced ejection fraction; LVEF: left ventricular ejection fraction; MR: mineralocorticoid receptor; MRA: mineralocorticoid receptor antagonist; T2D, type 2 diabetes; UACR, urinary albumin-to-creatinine ratio

The ongoing FIVE-STAR trial aims to investigate the effects of finerenone, relative to placebo, on vascular stiffness and relevant cardiorenal surrogate biomarkers in patients with T2D and CKD (Fig. 3). This study will help to provide information regarding the possible mechanisms underlying the clinical benefits of finerenone based on recent cardiorenal outcomes trials.

Despite the use of evidence-based pharmacological therapies (conventional RAS blockers and emerging SGLT2 inhibitors), the mortality and morbidity rates of T2D and CKD remain high, thereby indicating residual pathological targets that are not covered by existing medications. Finerenone, a novel nonsteroidal selective MRA, has been recently approved for treating patients with T2D and CKD based on its clinical benefits and safety according to the landmark cardiorenal outcome trials, FIDELIO-DKD [13], FIGARO-DKD [14], and the combined FIDELITY program [15]. Thus, an emerging and growing body of clinical evidence supports the promising therapeutic role of finerenone in achieving better clinical outcomes in patients with T2D and CKD.

In the previously mentioned clinical trials, the cardiorenal benefits of finerenone were theoretically driven by the suppression of MR overactivation, followed by anti-inflammatory and anti-fibrotic response in the kidney, heart, and vasculatures. Since finerenone can be widely use in clinical settings, its possible underlying mechanisms that mediate the aforementioned clinical benefits, should be explored in detail to help clinicians and researchers to better understand its clinical roles and cardiorenal actions. Considering the essential pharmacological action of finerenone, the early separation of the Kaplan–Meier curves for cardiovascular outcomes, and the delayed separation for renal outcomes in the aforementioned outcome trials [13, 14], the clinical benefits of finerenone might be, at least in part, mediated by a relatively immediate hemodynamic effect and gradual but persistent metabolic and tissue remodeling via the anti-inflammatory and anti-fibrotic signaling pathways derived from blocking MR overactivation. However, the detailed mechanisms underlying the mediation of the cardiorenal benefits of finerenone are not comprehensively examined in current clinical settings.

CAVI will be measured to assess the effect of finerenone on vascular stiffness, which is the primary endpoint of the FIVE-STAR trial. It is associated with cardiorenal function and the risk of adverse cardiorenal events in several cardiometabolic diseases, including diabetes and CKD [20, 24]. CAVI is independent of blood pressure at the time of measurement [17], and it has been used as a surrogate marker to determine the therapeutic impacts of cardiovascular and renal therapies in clinical trials [25]. However, there is currently little evidence on the effects of MRAs on CAVI. A pilot study revealed that eplerenone, a steroidal MRA, improved vascular endothelial function and arterial stiffness in patients with idiopathic hyperaldosteronism [26]. Thus, in our study, CAVI evaluation will potentially reflect the therapeutic impacts of finerenone on hemodynamic and vascular remodeling in patients with T2D and CKD.

In addition, serum and urinary biomarkers will be evaluated as secondary efficacy endpoints, thereby possibly providing practical evidence to monitor the cardiorenal efficacy of finerenone in clinical settings. Cardiac parameters, including left ventricular systolic and diastolic function, will also be measured on echocardiography. Although the trial will exclude symptomatic patients with HFrEF, patients with HF who have preserved ejection fraction (HFpEF) can participate in this trial. In our trial, evaluating the impact of finerenone therapy on cardiac function may be partly mutually related to the interpretation of an ongoing study to evaluate the efficacy and safety of finerenone in patients with HFpEF (FINEARTS-HF: NCT04435626) in the future. Furthermore, proteomics analysis using the Olink**®** Target 96 panels can explore the dynamic proteomics potentially involved in the pathways of finerenone treatment and can add novel mechanistic insights, as found in other MRA and SGLT2 inhibitors [27, 28].

SGLT2 inhibitor is recommended to reduce the risk of adverse cardiovascular and renal events in patients with CKD, irrespective of diabetes status [3, 7]. Moreover, SGLT2 inhibitor is currently recommended as an evidence-based initial medication for all patients with the HFpEF phenotype [29]. Recently, the effect of finerenone versus canagliflozin on the risk of cardiorenal events using data obtained from each corresponding outcome trial was comparable between agents in patients with T2D and CKD [30]. Thus, the clinical efficacy of finerenone is likely to similar to that of SGLT2 inhibitors. However, the mechanisms underlying the pharmacological action of SGLT2 inhibitors and finerenone differ. This finding may evoke a hypothesis that the dual use of finerenone and SGLT2 inhibitors have additive cardiorenal benefits in patients with T2D and CKD, and an exploratory study testing such hypothesis is ongoing (CONFIDENCE: NCT05254002). Due to a recent update in the relevant guidelines [3, 7, 29], some participants in the FIVE-STAR trial may already be receiving SGLT2 inhibitor therapy compared with those in the previous FIDELIO-DKD and FIGARO-DKD trials, thereby potentially enabling us to partly examine the hypothesis.

This trial may have several methodological limitations at the current stage. First, the study sample size is relatively small. Second, the study duration may be relatively short for evaluating changes in vascular stiffness and remodeling. Third, the number of evaluation visits for most efficacy endpoints of interest is limited and does not allow for serial evaluation of the therapeutic impact, especially immediately after treatment initiation. In addition, no association between the therapeutic impacts of finerenone on the study endpoints and subsequent long-term cardiorenal outcomes will be assessed in our study. Finally, although there are no eligible criteria regarding race and nationality, the current study will mainly include Japanese patients.

In conclusion, the FIVE-STAR trial is the first mechanistic clinical study that will examine the therapeutic effects of finerenone on vascular stiffness as assessed using CAVI and cardiorenal biomarkers in patients with T2D and CKD. This study will possibly provide a better understanding on the detailed mechanisms associated with the clinical benefits observed in previous cardiorenal outcome trials of finerenone.

## Supplementary Information

**Additional file 1. **Study organization of the FIVE-STAR trial.

**Additional file 2. **SPIRIT 2013 checklist.

**Additional file 3. **Dose adjustment criteria.

## Data Availability

Not applicable.

## Abbreviations

AE: Adverse event
AI: Augmentation index
CAVI: Cardio ankle vascular index
CI: Confidence interval
CKD: Chronic kidney disease
eGFR: Estimated glomerular filtration ratio
FAS: Full analysis set
HF: Heart failure
HFpEF: Heart failure with preserved ejection fraction
HFrEF: Heart failure with reduced ejection fraction
LVEF: Left ventricular ejection fraction
MR: Mineralocorticoid receptor
MRA: Mineralocorticoid receptor antagonist
%MAP: Percent mean arterial pressure
PWV: Pulse wave velocity
RAS: Renin-angiotensin system
SD: Standard deviation
SGLT2: Sodium glucose co-transporter 2
T2D: Type 2 diabetes
UACR: Urinary albumin-to-creatinine ratio
