# Supplementary material for: Rationale and design of an investigator-initiated, multicenter, prospective, placebo-controlled, double-blind, randomized trial to evaluate the effects of finerenone on vascular stiffness and cardiorenal biomarkers in type 2 diabetes and chronic kidney disease (FIVE-STAR)
Source: Cardiovasc Diabetol. 2023 Jul 31;22:194. doi: 10.1186/s12933-023-01928-y (PMC10391880; doi:10.1186/s12933-023-01928-y)
Supplement: Supplementary file 1 — Additional file 1. Study organization of the FIVE-STAR trial. [file 12933_2023_1928_MOESM1_ESM.docx]

**Additional file 1. Study organization of the FIVE-STAR trial**

**Principal investigator:**

Dr. Koichi Node (Saga University, Saga, Japan)

**Research Advisor:**

Dr. Hirotaka Shibata (Oita University, Yufu, Japan)

Dr. Masaomi Nangaku (The University of Tokyo Graduate School of Medicine, Tokyo, Japan)

**Study sites (chief investigator):**

1. Saga University (Dr. Koichi Node)
2. Oita University (Dr. Naohiko Takahashi)
3. Osaka Metropolitan University (Dr. Daiju Fukuda)
4. University of Occupational and Environmental Health, Japan (Dr. Yosuke Okada)
5. JR Hiroshima Hospital (Dr. Hiroki Teragawa)
6. Juntendo University Shizuoka Hospital (Dr. Satoru Suwa)
7. St. Marianna University School of Medicine (Dr. Keisuke Kida)
8. Toho University Ohashi Medical Center (Dr. Masao Moroi)
9. Dokkyo Medical University Saitama Medical Center, Saitama (Dr. Isao Taguchi)
10. Dokkyo Medical University School of Medicine (Dr. Shigeru Toyoda)
11. Fukushima Medical University (Dr. Michio Shimabukuro)
12. Mitsui Memorial Hospital (Dr. Kengo Tanabe)
13. Wakamatsu Hospital of the University of Occupational and Environmental Health (Dr. Kenichi Tanaka) Kitakyushu, Japan

**Study secretariat:**

Dr. Atsushi Tanaka (Saga University, Saga, Japan)

Ms. Keiko Onodera (The Organization for Clinical Medicine Promotion, Tokyo, Japan)

**Monitoring:**

Dr. Takuya Kishi (The Organization for Clinical Medicine Promotion, Tokyo, Japan)

**Data management:**

Mr. Kazushige Yoshimura (Translational Research Center for Medical Innovation, Kobe, Japan)

**Statistics:**

Mr. Takumi Imai (The Organization for Clinical Medicine Promotion, Tokyo, Japan)

**Randomization:**

Ms. Hisako Yoshida (The Organization for Clinical Medicine Promotion, Tokyo, Japan)

**Drug management:**

Mr. Kazuhisa Hosoya (Saga University Hospital)
