# Supplementary material for: Rationale and design of an investigator-initiated, multicenter, prospective, placebo-controlled, double-blind, randomized trial to evaluate the effects of finerenone on vascular stiffness and cardiorenal biomarkers in type 2 diabetes and chronic kidney disease (FIVE-STAR)
Source: Cardiovasc Diabetol. 2023 Jul 31;22:194. doi: 10.1186/s12933-023-01928-y (PMC10391880; doi:10.1186/s12933-023-01928-y)
Supplement: Supplementary file 3 — Additional file 3. Dose adjustment criteria. [file 12933_2023_1928_MOESM3_ESM.docx]

**Additional file 3. Dose adjustment criteria**

| **Serum potassium level (mEq/L)** | **Dose adjustment** |
| --- | --- |
| 4.8 or less  > 4.8 – 5.5 or less  > 5.5 | For patients treated with 10 mg once daily: increase to 20 mg once daily (only if the eGFR did not decrease by > 30% since the previous measurement)  For patients treated with 20 mg once daily: maintain the dose  Maintain the current dose  Discontinue the drug |

Based on the information in the package insert of finerenone.

*eGFR, estimated glomerular filtration rate.*
